# The effect of epigenetic silencing and TP53 mutation on the expression of DLL4 in human cancer stem disorder

**DOI:** 10.18632/oncotarget.11316

**Published:** 2016-08-16

**Authors:** Zhixing Yao, Zaki A. Sherif

**Affiliations:** ^1^ Department of Biochemistry & Molecular Biology, College of Medicine, Howard University, Washington DC, USA

**Keywords:** p53, DLL4, Notch, epigenetics, angiogenesis

## Abstract

The Li-Fraumeni Syndrome (LFS), a genetically rare heterogeneous cancer syndrome, is characterized *primarily* by a germline *p53* (*TP53*) gene mutation. We recently discovered a balanced reciprocal chromosomal translocation t(11;15)(q23;q15) in the non-cancerous skin fibroblasts of a bilateral breast cancer patient in LFS family. This prompted us to investigate the breakpoint region of the translocation, which uncovered a gene that encodes a Notch ligand, *DLL4*, (locus at 15q15.1), a key target in tumor vasculature. We analyzed *DLL4* gene expression and protein level in LFS non-cancerous skin fibroblast cell lines and non-LFS cancer cell lines. DLL4 is abrogated in all the LFS cells and drastically down-regulated in breast (MCF7) and brain (IMR32) cancer cells and tumor tissue samples. However, DNA methylation studies revealed that DLL4 promoter is silenced only in MCF7 but not in LFS cells. We further investigated the regulation of DLL4 gene expression by ChIP assays, which demonstrated that p53 binds to DLL4 promoter through its association with CTCF, a chromosomal networking protein CCCTC binding factor. This implies a possible karyotype-phenotype correlation with respect to DLL4 in LFS and breast cancer initiation and progression. The drastic reduction or absence in the expression of DLL4 in LFS as well as breast and brain cancer cells is significant and supports the concept that this ligand may also play a role in cancer immune-surveillance; and its resuscitation in the tumor microenvironment may stimulate T-cell immunity and suppress tumor growth. Therefore, DLL4 may provide a strong platform as an immuno-therapeutic target in LFS and cancer patients.

## INTRODUCTION

The Li-Fraumeni syndrome (LFS; OMIM #151623) is a clinically and genetically heterogeneous cancer syndrome with inherited germline heterozygous mutation in the tumor suppressor gene, p53 (*TP53*), which can provide powerful insights into our understanding of the somatic mutations present in sporadic cancers. Widespread as an acquired anomaly in cancer, the role of *TP53* as a germline mutation particularly in LFS continues to unfold [1, 2]. TP53 is touted as the guardian of the genome because it counteracts the activation of oncogenes whose clinical impact is often disastrous [3, 4].

The Notch signaling pathway is instrumental in mammalian development, cell fate determination, differentiation, proliferation, angiogenesis and survival in eukaryotes [5–9]. It also plays an important role in tumor suppression through inhibition of proliferation, differentiation and apoptosis in multiple cell types [10–12]. Its core function is mediated by the human delta- like ligand-4 (DLL4) gene that encodes a membrane-bound protein for the Notch family of receptors [13]. DLL4, one of four delta-like ligands, is a highly conserved 685 amino acid single- pass type I transmembrane heterodimer protein whose extracellular unit contains epidermal growth factor-like repeats, glycosylation sites and a DSL (Delta,/Serrate,/Lag2) domain that is important for interaction with Notch receptors (N1-N4). Although DLL4 can be expressed in brain, retina, thymus and hematopoietic cells, it is primarily expressed in endothelial cells of developing vessels and sprouting blood vessels [14].

Notch signaling is routinely activated by proteolysis via gamma-secretase and its regulation is controlled by the posttranslational modification of ligands and receptors. A recent *Nature* paper, Guarani et al. [15] reported that Notch signaling can be negatively controlled by SIRT1, a protein deacetylase that works by deactivating the Notch-1 intracellular domain and thereby blocking transcriptional regulation of nuclear target genes. Dysregulation of this highly conserved pathway may lead to the genesis of many human cancers such as T-Cell Acute Lymphoblastic Leukemia and several developmental syndromes by thwarting cell-fate determination during development and compromising tissue homeostasis [16–23]. The dysregulation of the DLL4/Notch signaling pathway has also been linked to many types of metastatic cancer [5],[24–26]. VEGF, located upstream of DLL4, is involved in a feedback loop with DLL4, which negatively regulates VEGF-mediated angiogenesis [27]. However, several anti-VEGF drugs have met with tumor resistance in long-term treatment regimens and preclinical studies involving adjuvant anti-VEGF therapies may actually augment the risk of metastasis [28]. Similarly, blockade of DLL4-Notch signaling for example using gamma-secretase inhibitors has been associated with dramatically enhanced endothelial cell proliferation and increased expression of VEGFR2 and VEGFR3; and causing complete obstruction of T-cell development [29–31]. Chronic blockade of DLL4 with antibodies induces vascular tumorigenesis in animals; and Notch inactivation or mutation also causes vascular tumors in mice [7, 32], [33, 34].

Recent studies also report that Notch activation is evident during the overexpression of *DLL4* resulting in the reduction of tumor growth and vascularization [27, 35]. Furthermore, Notch-1 has been identified as a tumor suppressor in mouse prostate and skin [12, 28, 36]. The clinical value of Notch signaling is that it regulates both apoptosis and proliferation in hematopoietic systems [37]. Therefore, according to published reports cited above, Notch acts like a molecular switch by promoting measured angiogenesis during normal development and blocking aberrant tumorigenesis during pathological conditions.

We previously showed the presence of a novel balanced reciprocal translocation between chromosomes 11q23 and 15q15 in an LFS breast cancer patient's normal skin fibroblasts [38]. Analysis of the DNA in the breakpoint regions identified a number of genes, chiefly *DLL4*. We investigated the role of DLL4 and its potential contribution to carcinogenesis and tumorigenesis in LFS as well as cancer cell lines and corresponding tumor tissues. We also examined biochemical interaction between TP53 and DLL4 promoter to identify a potential regulatory role. Our findings establish that reduced expression or dysregulation of DLL4, in addition to *TP53* mutation, is a key mechanism for Notch-mediated predisposition to carcinogenesis and tumorigenesis in LFS. This study is an attempt to contribute to the rational design of potent clinical therapeutics by characterizing DLL4 protein expression in human cancer.

## RESULTS

### DLL4 gene expression in Li-Fraumeni syndrome cell lines and cancer cell lines

**Figure 1:**
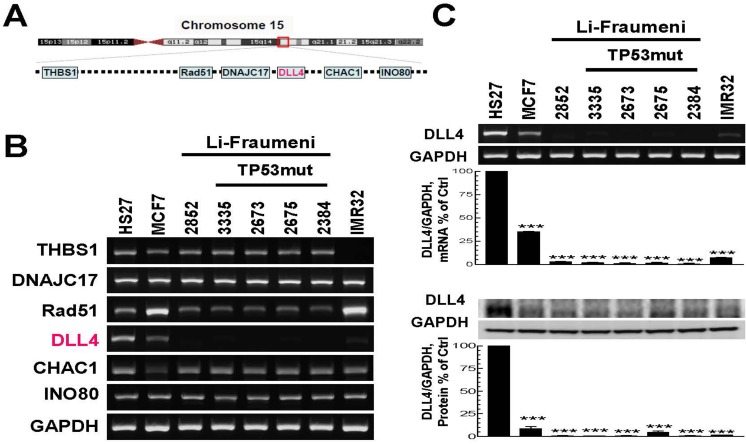
Analysis of gene expression in the breakpoint region of chromosome 15q15 and determination of the corresponding protein levels in the normal skin fibroblasts of Li-Fraumeni Syndrome (LFS) patients and unrelated cancer cell lines **A.** A schematic representation of several genes located in chromosome 15q15. **B.** mRNA levels of the genes located in chromosome 15q15 in LFS cell lines and breast cancer cell line, MCF7 as well as neuroblastoma cell line IMR32, compared with normal human foreskin fibroblast cell line, HS27. **C.** DLL4 mRNA and corresponding protein levels in LFS and other cancer cell lines displayed in B. RT-PCR (upper panel) and immunoblot (lower panel) showing decreased expression of DLL4 in LFS cell lines, MCF7 as well as IMR32, in contrast to normal human foreskin fibroblast cell line, HS27. Densitometric analyses and the results shown in panel C reflect a mean ±S.E.M. from three independent experiments, performed in triplicates. Significance: ***P < 0.001 compared with control values, determined by *t*-test.

Due to a balanced reciprocal translocation between chromosomes 11q23 and 15q15 in LFS patient's cells [38], we examined the gene expression levels of *THBS1, DNAJC17, Rad51, DLL4, CHAC1 and INO80* located in chromosome15q15 region (Figure 1A) by RT-PCR (Figure 1B). Surprisingly, the results show that the expression of most of these genes and especially DLL4, is dysregulated in LFS cell lines as well as in breast cancer cell line (MCF7) and neuroblastoma cell line (IMR32) when compared to normal foreskin human fibroblast cell line, HS27. We further analyzed the DLL4 mRNA and protein levels in LFS cell lines and cancer cell lines by using RT-PCR (Figure 1C, upper panel) and immunoblot (Figure 1C, lower panel). The results confirmed that the expression of Delta-like ligand 4 (DLL4) is abrogated in normal skin fibroblasts (NSFs) of LFS family regardless of their status as wild-type (WT) *TP53* carriers or mutation (MT) carriers. The results also show marked down-regulation in the expression of DLL4 in breast cancer cell lines and neuroblastoma cell lines when all are compared to a normal fibroblast cell line, HS27, used as control. Figure 1C also displays analysis by densitometry and the percentage of DLL4 expression in each cell line as per the control in triplicate samples at both the mRNA and protein levels.

### DLL4 gene expression in human tumor tissues and matched normal tissues

**Figure 2:**
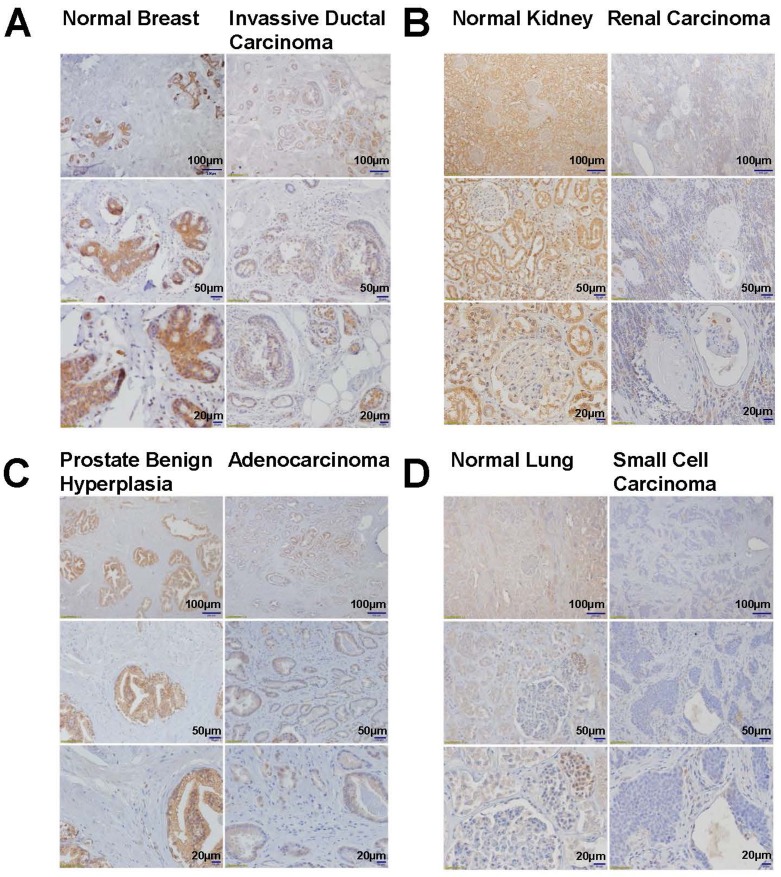
Immunohistochemistry analyses of DLL4 expression in normal, benign hyperplasia and tumor tissues **A.** Breast, **B.** Kidney, **C.** Prostate, **D.** Lung.

**Figure 3:**
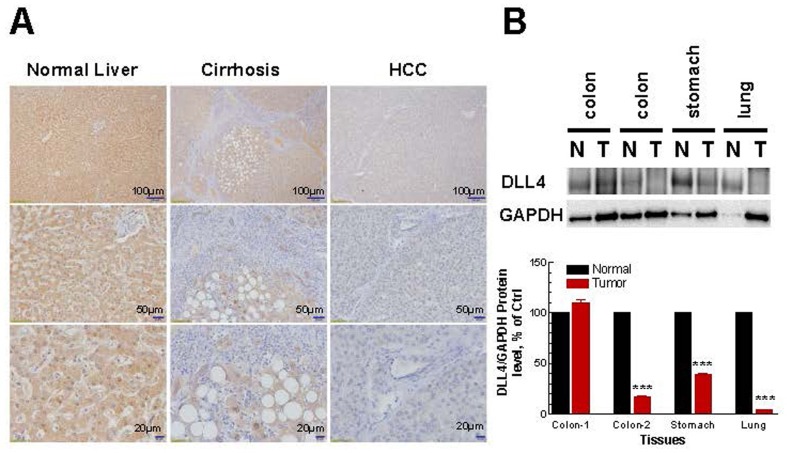
Immunohistochemistry and densitometric analyses of DLL4 expression in normal, cirrhotic and tumor tissues **A.** Immunohistochemistry analyses of DLL4 expression in normal, cirrhotic and tumor liver tissues. **B.** Immunoblot analyses of human normal tissue and tumor tissue samples including colon, stomach and lung. Densitometric analyses and the results shown in lower panel reflect a mean ±S.E.M. from three independent experiments, performed in triplicates. Significance: ***P < 0.001 compared with control values, determined by *t*-test.

To confirm that the findings of DLL4 down-regulation in cancer cell lines was not just a genetic phenomenon restricted only to the cell lines, we analyzed the DLL4 protein level in normal and cancer patients’ tumor tissues by immunohistochemistry (IHC). First of all, the DLL4 antibody used for IHC in Figures 2 and 3 has been validated. As shown in Figure 2, the results similarly indicate that DLL4 was down-regulated in invasive ductal carcinoma (Figure 2A), renal carcinoma (Figure 2B), prostate adenocarcinoma (Figure 2C) and lung small cell carcinoma (Figure 2D) all in contrast to their corresponding normal tissues or benign hyperplasia (prostate). Moreover, the immunohistochemical (IHC) staining also shows that DLL4 expression is slightly decreased in human liver cirrhotic tissues and highly down-regulated in hepatocellular carcinoma (HCC) in comparison to normal liver tissue (Figure 3A). Additionally, immunobolt results revealed that DLL4 expression in human colon, stomach and lung tumor tissues is dramatically down-regulated as compared to normal tissue (Figure 3B). Interestingly, in all these various types of tumor tissues, the DLL4 expression was significantly reduced compared to their normal tissue counterparts. The analysis of paired normal and tumor samples may seem to be limited by the number of cases due to high background (e.g., two different results in the two different colon tumor cases).

### DLL4 is silenced at its promoter site by DNA methylation and reactivated by the inhibitor of DNA methylation in breast cancer cell line

**Figure 4:**
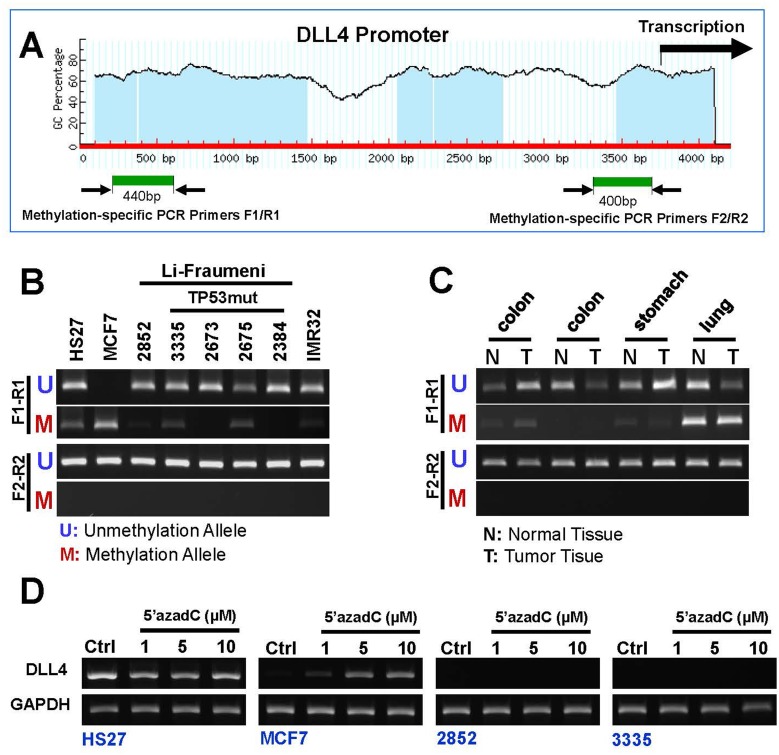
DNA methylation pattern of DLL4 gene promoter in LFS, breast and brain cancer cell lines **A.** Schematic representation of DLL4 promoter and CpG islands; **B.** Methylation status of the DLL4 promoter in LFS cell lines and cancer cell lines as detected by MS-PCR; **C.** Methylation status of the DLL4 promoter in human normal tissue and tumor tissue samples including colon, stomach and lung. **D.** Marked Reactivation of DLL4 gene expression in MCF7 but neither in the two DLL4-negative LFS cell lines nor in the normal cell line, HS27 by the DNA methylation inhibitor, 5-aza-dC.

We know that these simple expression studies do not prove functional activity in and of themselves. We therefore carried out an epigenetic study. Growing evidence suggests that aberrant DNA methylation of CpG islands around promoter regions can have the same effect as coding region mutations or on inactivation of tumor-suppressor genes [39]. Since the promoter region of DLL4 contains a typical CpG island (Figure 4A), we examined the methylation state in genomic DNA isolated from eight cell lines (five LFS cell lines, one breast cancer cell line (MCF7), one neuroblastoma cell line (IMR32), and normal human foreskin fibroblast cell line HS27), utilizing methylation-specific PCR (Figure 4B). As the results confirm, only the DLL4 promoter in human breast cancer cell line (MCF7) shows a positive correlation between low-level of DLL4 expression and the methylation state in the distal of promoter region and not in the proximal of promoter region (Figure 4B). Moreover, methylation-specific PCR also revealed that CpG island DNA methylation at distal promoter region in human colon, stomach and lung tumor tissues and normal tissue (Figure 4C). 5′-aza-2′-deoxycytidine (5′-aza-dC), an inhibitor of DNA methylation, can reactivate gene expression when hypermethylation of CpG islands is the cause of reduced gene expression [40]. To demonstrate regulation of DLL4 expression by DNA methylation, two LFS cell lines, a TP53 mutation carrier (3335) and a non-carrier (2852) of the cancer-prone family under study, breast cancer line (MCF7) and a normal control (HS27) were treated with 5′-aza-dC for 4 days. As shown in Figure 4D, 5 μM of 5′-aza-dC reactivated DLL4 expression in MCF7 but neither in the two DLL4-negative LFS cell lines nor in the normal cell line, HS27. These results demonstrate that DNA methylation is not the cause of promoter silencing or abrogation of DLL4 expression in LFS cells.

### CTCF and TP53 are involved the regulation of DLL4 gene expression

**Figure 5:**
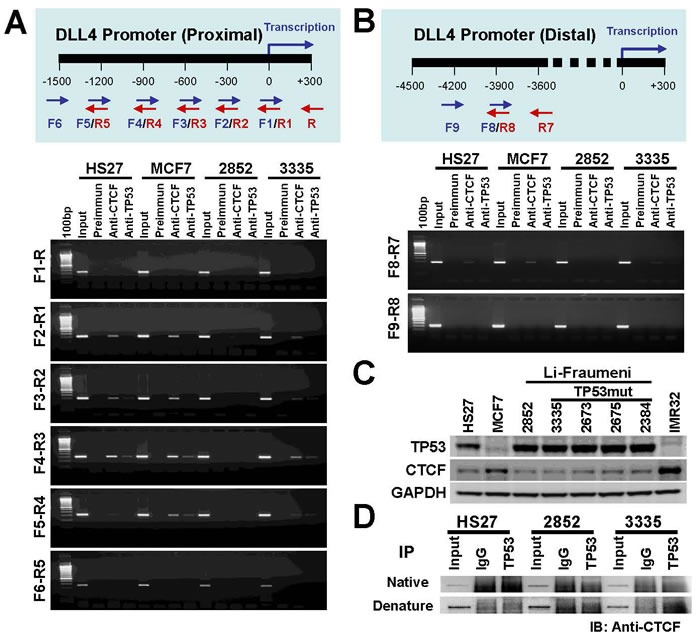
Role of TP53 and CTCF in regulation of DLL4 gene expression **A.** Ideogram representing primers used in this ChIP assay for DLL4 proximal promoter region (upper panel) and regulation of DLL4 gene expression in LFS and MCF7 cells by ChIP assay. HS27 cells were used as control (lower panel). **B.** Ideogram representing primers used in this ChIP assay for DLL4 distal promoter region (upper panel) and regulation of DLL4 gene expression in LFS and MCF7 cells by ChIP assay in DLL4 proximal promoter region. HS27 cells were used as control (lower panel). **C.** TP53 and CTCF protein levels in LFS, MCF7 and IMR32 cancer cell lines. **D.** Determining the interaction between TP53 and CTCF by co-immunoprecipitation.

CTCF is a chromosomal networking protein CCCTC binding factor and a key regulator and repressor of IGF2 [41]; as a transcriptional insulator element, CTCF can block communication between enhancers and upstream promoters, thereby regulating imprinted expression [42]. We investigated the regulation of DLL4 gene expression by ChIP assay using primers represented in Figure 5A (upper panel) for proximal promoter region. The results demonstrate that the association of TP53 with CTCF may involve DLL4 gene regulation through the binding of TP53 to DLL4 promoter (Figure 5A lower panel). The CHIP assays at the proximal promoter region also show that DLL4 expression in LFS cells is mostly dysregulated when compared to the normal cell, HS27, and breast cancer cell, MCF7, possibly due to TP53 mutation or functional alterations that predispose to abnormal genetic consequences. We also examined the DLL4 distal promoter region by ChIP assay using primers represented in Figure 5B (upper panel). The results showed that TP53 and CTCF were involved the regulation of DLL4 gene expression (Figure 5B lower panel). Considering that TP53 and CTCF protein levels may alter DLL4 gene expression, we measured TP53 and CTCF protein levels in the different cell lines by immunoblotting (IB) (Figure 5C). The resulting data show that TP53 was up-regulated in LFS cell lines even relative to the normal cell line, HS27, and down-regulated in MCF7 and IMR32 cancer cell lines. In contrast, the protein level of CTCF was not markedly changed in LFS cell lines but is significantly up-regulated in MCF7 and IMR32 cancer cell lines in comparison to normal cell, HS27. Moreover, to determine the interaction between TP53 and CTCF, we performed a co-immunoprecipitation assay under native conditions (without detergent) and denaturing conditions (with detergent). The result shows that TP53 does not bind to CTCF under either condition (Figure 5D).

**Figure 6:**
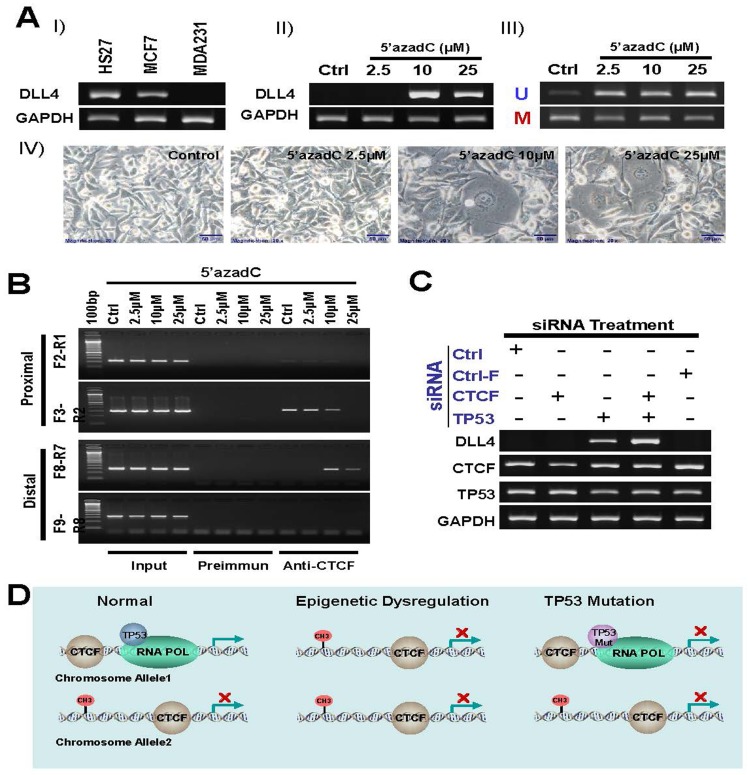
Role of DNA methylation, TP53 and CTCF in regulation of DLL4 gene expression **A.** silenced DLL4 by DNA methylation in MDA231 cell line is reactivated by inhibitor of DNA methylation 5′-aza-dC. I) DLL 4 expression level in MDA231 cell line is compared with HS27 and MCF7 by RT-PCR assay. II) Reactivation of silenced DLL4 gene in MDA231 by the DNA methylation inhibitor, 5-aza-dC. III) Methylation status of the DLL4 promoter in MDA231 cell lines treated with 5-aza-dC and detected by MS-PCR. IV) Phase-contrast photomicrographs showing morphologic changes in MDA231 cells treated with 2, 5μM, 10μM and 25μM of 5-aza-dC for 3 days, compared with untreated control cells maintained for 3 days. **B.** The effect of DNA methylation on interaction between CTCF and DLL4 promoter by ChIP assay **C.** Reactivation of DLL4 gene expression in LFS cell line, 3335, by CTCF and TP53 siRNA treatment. **D.** A schematic representation of presumable mechanism of regulation of DLL4 gene expression.

To investigate the details of an association between DNA methylation and CTCF in DLL4 gene regulation, we treated another breast cancer cell line, MDA231, which has a silenced DLL4 gene expression (Figure 6A-I), with increasing concentrations of 5′-aza-dC for 3 days. The results show that the DNA methylation inhibitor, 5′-aza-dC at 10μM concentration, reactivated the expression of DLL4 in MDA231 cell line (Figure 6A-II). The additional effect of 5′-aza-dC on the DNA methylation state of the DLL4 distal of promoter in MDA231 cell line is shown in Figure 6A-III and the effect of 5′-aza-dC on MDA231 cell morphology is shown in Figure 6A-IV. We further determined the effect of DNA methylation on interaction between CTCF protein and DLL4 promoter by ChIP assay. The results reveal that 5′-aza-dC treatment (2.5-25 μM) led to dramatically enhanced CTCF protein binding to the distal of DLL4 promoter region and a marked decreased binding to proximal promoter region (Figure 6B). These data indicate that DNA methylation of DLL4 in the distal promoter region may be responsible for CTCF binding to DLL4 proximal promoter region that prevented DLL4 gene transcription.

To determine the role of TP53 mutation in DLL4 gene regulation, we treated the LFS cell line, 3335, with CTCF and TP53 siRNAs. Surprisingly, the results demonstrate that DLL4 is not expressed in the presence or absence of CTCF alone but is reactivated by the knockdown of TP53 and even more strongly during the knockdown of both TP53 and CTCF as shown in lane 4 (Figure 6C). These observations suggest that TP53 in coordination with CTCF plays a key role in DLL4 gene expression even though there is no direct interaction between TP53 and CTCF. A schematic representation of presumable mechanism of regulation of DLL4 gene expression is shown in Figure 6D.

## DISCUSSION

DLL4/Notch signaling is a tightly coordinated system of checks and controls that balances the need for growth through the expansion of the vascular endothelial cells against the danger of overgrowth by sprouting endothelial tip cells. Notch signaling is not only important for cellular differentiation in multi-cellular organisms but it is also crucial for many aspects of early T-cell development. But in the case of LFS cells where a chromosomal translocation was discovered in a homozygous wild-type p53-containing patient, the DLL4 abrogation cannot be explained by Notch activity alone. In fact, if there is a disruption of the DLL4 gene because of its precise location in the breakpoint region of the translocation, a reduction in Notch expression would have been expected. However, Notch expression was unaltered in all the LFS cell lines although its activity was not measured.

The precise and expansive roles of Notch and DLL4 and their relationship with TP53 in LFS and tumorigenesis are poorly understood and this is the focus of this study. Why was DLL4 abrogated in all the LFS cell lines regardless of their TP53 status? Moreover, unlike some published reports, the results also show drastic down-regulation of DLL4 in cancer cell lines and all the tumor samples examined. The abrogation of DLL4 expression in LFS is a novel discovery, which to our knowledge has never been reported before, especially in light of the fact that the main characteristic feature of LFS has always been the germline and autosomal dominant heterozygous mutation of *TP53*. However, a similar down-regulation of DLL4 both at the transcriptional and translational levels in other cancer lines and tumor tissues was unexpected and antithetical to published reports that emphasize DLL4 as a tumor promoter and an aberrant activator of the Notch receptor. This has prompted the development of anti-DLL4 antibody and gamma secretase inhibitors as therapeutic agents for cancer patients. These efforts have not been very successful and raise concerns for long-term treatment of cancer using DLL4 and Notch inhibitors. Our results, on the other hand, point to a potential contribution of a suppressed or dysregulated DLL4 to tumorigenesis. Therefore, exclusive reliance on anti-DLL4 therapy may have long-term and undesirable effects.

According to the results in our study, epigenetic regulation via DNA methylation of the DLL4 promoter sites was not the cause of DLL4 abrogation in these LFS cells, although silencing through methylation might have been a factor in MCF7 cells, which only exhibited reduction of DLL4 expression and not a total absence of the gene or the protein. What is most intriguing in our findings is the total absence of DLL4 expression in normal (non-cancerous) skin fibroblasts of wild-type *TP53* carriers and mutant *p53* carriers of LFS patients who had a preponderance of primary cancers at an early age. It was therefore necessary to investigate the presence of an interaction between DLL4 and TP53, whose germline mutation is the hallmark of the Li-Fraumeni syndrome. Although the results indicate a possible physical association between TP53 and CTCF as a requirement in the regulation of the expression of DLL4, the ChIP assay and the siRNA experiments in this study clearly show the presence of a binding site for TP53 on the DLL4 promoter. This is the first reported case as far as we know of a transcriptional regulation of DLL4 by the tumor suppressor protein, TP53. The exact role of CTCF's association with TP53 in regulating DLL4 expression is not known. However, CTCF is a chromosomal networking protein CCCTC binding factor and a key regulator and repressor of IGF2; and as a transcriptional insulator element, it can block communication between enhancers and upstream promoters [41, 42]. The question still remains as to why DLL4 is abolished in LFS cells under both wild-type and mutant p53 conditions. As Figure 5C shows, there is no difference in the protein expression of wild-type TP53 and mutant TP53 in LFS cells suggesting that the wild-type allele activity of TP53 in LFS cells may not be as potent in its tumor suppression ability especially in curbing cell proliferation as wild-type TP53 found in normal non-LFS cells [43]. Furthermore, not surprisingly, the gain of function ability of the dominant negative mutant TP53 in a heterozygous configuration might block DLL4 expression. This might be the reason that knocking down *TP53* in a heterozygous mutant LFS cell line, 3335, by siRNA reactivated DLL4 expression. We have not determined the potential presence of haploinsufficiency in the DLL4 gene in any of our experimental samples.

As shown in this study, the down-regulation of DLL4 is also drastic in tumor samples derived from breast, brain, kidney, lung, liver, and prostate. One possibility, although not conclusively shown, is the presence of gene disruption in the region of DLL4 where the chromosomal translocation has occurred. More importantly, the dysregulation of DLL4 seems to be common in all cells including LFS and tumor tissues. However, during the initiation of Notch signaling, a transcriptional coactivator is released when DLL4 interacts with a Notch receptor on an adjacent cell and activates the receptor through proteolysis. This active intracellular domain may also impinge on the transactivation of TP53 function and thus inhibit the expression of downstream genes that regulate apoptosis, senescence or cell cycle arrest [44]. This suggests a possible feedback loop between TP53 and Notch mediated by DLL4. Activated Notch-1 plays a dichotomous role by inducing cell cycle arrest and terminal differentiation via p21 regulation on one hand, and on the other hand, by transcriptional inhibition of genes involved in neuronal differentiation [44]. These Notch-targeted processes with dual roles are very important in the context of the influence of DLL4/Notch signaling in tumorigenesis.

The core functional component of the Notch signaling pathway is DLL4. It has been observed that mice devoid of DLL4 expression in thymic epithelial cells showed a drastic reduction of Notch-1 in hematopoietic cells and a lack of Cd4 and Cd8 double- or single-positive T-cells in thymus. This demonstrates the importance of the intracellular fragment of Notch-1 for T-cell progenitor generation and thymic T-cell differentiation as both functions were able to be restored by forced expression of the intracellular domain [10, 11]. This also suggests that for the induction of Notch signaling in cells migrating into the thymus, DLL4 expression is paramount [45]. It has also been reported that actively growing tumors down-regulate the expression of DLL1 and DLL4 especially in hematopoietic cells under circumstances in which immune-surveillance (i.e. T-cell activation) is downgraded and angiogenesis is promoted [46].

In this study, the negative expression of DLL4 in LFS and cancer cells was not restricted to only tissue culture cell lines but it was also evident in tumor tissues from invasive ductal carcinoma, renal carcinoma, prostate adenocarcinoma, small cell carcinoma, hepatocellular carcinoma, and neuroblastoma. This suggests that a dysregulated DLL4 can contribute to a widespread carcinogenesis or tumorigenesis if its expression and function in these cells are compromised. Although the underlying cause of DLL4 dysregulation in LFS or other cancer cell lines and tissues may not be fully known, our results suggest that 1) the presence of a germline TP53 mutation or 2) the prevalence of epigenetic conditions or 3) even the contribution of a gene-disruption by translocation, whose presence may be interfering with the normal function of the immune system, may be partially or wholly responsible for the subsequent development of tumorigenesis.

Our data showed that TP53 and CTCF together have a direct activator effect at the DLL4 promoter. CTCF, potentially, is a key regulator and repressor/activator of DLL4. However, the mechanisms by which the CTCF/TP53 axis is regulated by signaling are not understood. Defining the context of DLL4 expression is critical to our understanding of potential treatment options available to cancer patients. Our observation of association of TP53, CTCF and DLL4 in Notch signaling provides a mechanism by which significant developments can be achieved for future therapeutic applications.

## MATERIALS AND METHODS

### Human LFS cell lines and cell culture

LFS cells were grown at 37°C, 5% CO_2_, and in minimum essential medium with Earl's salts and L-glutamine (Life Technologies, Bethesda, MD) containing 10% fetal bovine serum and 25 mmol/L HEPES. Cells underwent low passages and were harvested at 75-90% confluence [38]. MCF7 and IMR32 cell lines were obtained from ATCC (Manassas, VA) and cultured following the instructions of the supplier.

### DNA methylation analysis

Genomic DNA was bisulfite-modified with an EpiTect Bisulfite Kit (Qiagen, CA, USA) according to the manufacturer's protocols. Prediction of CpG islands in β2SP promoter and primer design for methylation-specific PCR use were obtained through a web software (www.urogene.org); Primer pairs used for DLL4 distal promoter region methylation-specific PCR were methylated forward / 5′- TTA TTG ATC GGT AGG TGC GAG TAG C -3′ reverse/ 5′- CAC GTA CAA AAA ACG ACG ACC G -3′ and unmethylated forward 5′- TTG ATT TAT TGA TTG GTA GGT GTG AGT AGT -3′; reverse/ 5′- AAA ACC ACA TAC AAA AAA CAA CAA CCA -3′. Primer pairs used for DLL4 proximal promoter region methylation-specific PCR were methylated forward / 5′- GAA AAG GAG ATC GGA TTT CCC TAG C -3′ reverse/ 5′- TCT AAC TAC TAC AAT CCC AAC GCC G -3′ and unmethylated forward 5′- AGG AAG GAA AAG GAG ATT GGA TTT TTT TAG T -3′; reverse/ 5′-CCT CTA ACT ACT ACA ATC CCA ACA CCA -3′.

### RT-PCR and immunoblotting (IB)

The primers used for DLL4 amplifications were as follows: forward/ 5′-GGG ATG GCG GCA GCG TCC -3′; reverse/ 5′-TAC CTC CGT GGC AAT GAC ACA TT CA -3′. Rabbit anti-DLL4 (Cat#2589) for IB from Cell Signaling Technology (Boston, MA); Human normal tissue and tumor tissue samples including colon, stomach and lung were kindly provided by Dr. Edward Lee of the Department of Pathology, Howard University Hospital.

### siRNA treatment

Briefly, CTCF, TP53 (Santa Cruz; sc-35124 and sc-44218) and non-silencing control as well as fluorescein-conjugate (Santa Cruz, sc-37007 and sc-36869) were used at 60 nM to transfect LFS cell line, 3335, using siRNA Reagent System (Santa Cruz, sc-45064) in serum-free media for 6h according to manufacturer's instructions, Knockdown and transfection efficiency of siRNAs were confirmed by RT-PCR and Fluorescence Microscopy. The primers used for CTCF and TP53 amplifications were as follows: forward/ 5′-GAA ATG GAA GGT GAT GCA GTC GAA GC -3′, reverse/ 5′- CCG GTC CAT CAT GCT GAG GAT CA -3′; and : forward/ 5′- GCC ATG GAG GAG CCG CAG TCA-3′, reverse/ 5′-TCA GTC TGA GTC AGG CCC TTC TGT CTT-3′.

### Immunohistochemistry (IH)

Immunohistochemistry was performed using validated antibody against DLL4 at the Lombardi Comprehensive Cancer Center Histopathology & Tissue Shared Resource, Georgetown University Medical Center. Briefly, immunohistochemical staining of normal and tumor tissue samples of breast, kidney, liver, lung and prostate was performed for human DLL4 made in rabbit. Five micron sections from formalin fixed paraffin embedded tissues were de-paraffinized with xylenes and rehydrated through a graded alcohol series. Heat induced epitope retrieval (HIER) was performed by immersing the tissue sections at 98°C for 20 minutes in 10 mM citrate buffer (pH 6.0) with 0.05% Tween. Immunohistochemical staining was performed using a horseradish peroxidase labeled polymer #K4003 (Dako North America, Carpinteria, CA) according to manufacturer's instructions. Briefly, slides were treated with 3% hydrogen peroxide and 10% normal goat serum for 10 minutes each and exposed to primary antibody DLL4 (1:60, Abcam, Cat # ab176876 ) diluted in 1X TBS with 0.05% Tween 20 (Fisher, Pittsburg, PA) for overnight at 4°C. Slides were exposed to the HRP labeled polymer for 30min and DAB chromagen (Dako) for 5 minutes. Slides were counterstained with Hematoxylin (Fisher, Harris Modified Hematoxylin), blued in 1% ammonium hydroxide, dehydrated, and mounted with Acrymount. Consecutive sections with the primary antibody omitted were used as negative controls. Wash buffer used 1X TBS with 0.05% Tween 20 (Fisher).

### Co-immunoprecipitation (Co-IP) and chromatin-immunoprecipitation (ChIP) assays

Co-immunoprecipitation assay was performed using Pierce Classic IP Kit according to manufacturer's instructions (Thermo-Scientific, Waltham, MA) Rabbit anti-TP53 (Cat#2527) for Co-IP and Rabbit anti-CTCF (Cat#3418) from Cell Signaling Technology (Boston, MA). ChIP assay was performed using ChIP assay kit according to manufacturer's instructions (Upstate Biotechnology, Lake Placid, NY). Rabbit anti-CTCF and TP53 for ChIP is the same as Co-IP. Primers for DLL4 proximal promoter were as follows: forward/ F1) 5′- CAG GTT TCA GTA GCG GCG CTG -3′; F2) 5′- ATT ACC GGG CAA CCC CTC TAT CC -3′; F3) 5′- GAG TGG CCA CAG AGA GGT TAAC GC -3′; F4) 5′- CGC AGG AAC TGA AGC TGG ACT C -3′; F5) 5′- GAT CAC GCC GGG TTC CGA GAA -3′; F6) 5′- AAC CCA CGC TCC CAA CCT CTT -3′. Reverse / R) 5′- GGA CGC TGC CGC CAT CC -3′; R1) 5′- CAG CGC CGC TAC TGA AAC CTG -3′; R2) 5′- GGA TAG AGG GGT TGC CCG GTA AT -3′; R3) 5′- GCG TTA ACC TCT CTG TGG CCA CTC -3′; R4) 5′- GAG TCC AGC TTC AGT TCC TGC G -3′; R5) 5′- TTC TCG GAA CCC GGC GTG ATC -3′. Primers for DLL4 distal promoter were as follows: forward/ F8) 5′-CTT GAA ACT GCG GCG CCT GAA T-3′; F9) 5′-CCA GAG AGA GGT GAA GGA GGC CAC-3′. Reverse / R7) 5′- GAG AAG GGG CCA CGT GCA GG-3′; R8) 5′- ATT CAG GCG CCG CAG TTT CAA G-3′.

### Statistics

Statistical analysis was performed by one-way analysis of variance (ANOVA) and unpaired Student's t test using the INSTAT 3.00 package (GraphPad, San Diego, CA, USA).
